# Review on Facial-Recognition-Based Applications in Disease Diagnosis

**DOI:** 10.3390/bioengineering9070273

**Published:** 2022-06-23

**Authors:** Jiaqi Qiang, Danning Wu, Hanze Du, Huijuan Zhu, Shi Chen, Hui Pan

**Affiliations:** 1 Key Laboratory of Endocrinology of National Health Commission, Department of Endocrinology, Peking Union Medical College Hospital, Chinese Academy of Medical Sciences and Peking Union Medical College, Beijing 100730, China; qiangjiaqi@student.pumc.edu.cn (J.Q.); danie_wu@student.pumc.edu.cn (D.W.); duhanze@pumch.cn (H.D.); zhuhj@pumch.cn (H.Z.); 2 Eight-Year Program of Clinical Medicine, Chinese Academy of Medical Sciences and Peking Union Medical College, Beijing 100730, China; 3 State Key Laboratory of Complex Severe and Rare Diseases, Peking Union Medical College Hospital, Chinese Academy of Medical Sciences and Peking Union Medical College, Beijing 100730, China

**Keywords:** facial recognition, disease diagnosis, artificial intelligence, automated identification

## Abstract

Diseases not only manifest as internal structural and functional abnormalities, but also have facial characteristics and appearance deformities. Specific facial phenotypes are potential diagnostic markers, especially for endocrine and metabolic syndromes, genetic disorders, facial neuromuscular diseases, etc. The technology of facial recognition (FR) has been developed for more than a half century, but research in automated identification applied in clinical medicine has exploded only in the last decade. Artificial-intelligence-based FR has been found to have superior performance in diagnosis of diseases. This interdisciplinary field is promising for the optimization of the screening and diagnosis process and assisting in clinical evaluation and decision-making. However, only a few instances have been translated to practical use, and there is need of an overview for integration and future perspectives. This review mainly focuses on the leading edge of technology and applications in varieties of disease, and discusses implications for further exploration.

## 1. Introduction

The face is a unique marker of bioidentity for humankind. It provides information in regards to age, sex, race, consciousness, emotion, and health status. As it is conveniently accessible and cost-effective, the face is widely accepted for reliable biometrics compared with the fingerprint and iris [1,2]. Various diseases manifest not only as internal structural and functional abnormalities, but they also have facial characteristics and deformities. Diseases with facial manifestations are mainly endocrine and metabolic disorders [3], genetic syndromes [4], and neuromuscular diseases [5], some of which are complex and rare diseases. Early diagnosis and differentiation of these diseases are essential for timely therapy and better prognosis. To identify typical facial features is a part of the traditional diagnosis path, and largely depends on expertise and experience.

Automatic technology for facial recognition appeared in the 1960s, and mature approaches have been developed in real-world applications, covering areas of security surveillance, identity verification, forensic science, law enforcement, etc. [1]. Pioneer research on facial recognition applications in disease diagnosis dates back to the 2000s. Children’s genetic syndromes [6,7] and facial neuromuscular dysfunction [8] were the first diseases detected by knowledge-based methods. In recent years, the emergence of artificial intelligence (AI) has changed human life and has also led to breakthroughs in healthcare. Medical image analysis is the most rapidly developed domain in AI medicine, and broad progress has been made in radiology, pathology, ophthalmology, dermatology, and gastroenterology [9,10]. Facial recognition, as an essential part of automatic image analysis, also displays outstanding efficacy in the era of AI. The number of publications has presented exponential growth since the year 2013. Facial recognition has been introduced at a larger scale to assist diagnosis of diseases that feature facial abnormalities. More disease varieties and racial groups have been incorporated into this dynamic division of AI medicine.

As automated image-based diagnosis is becoming mature, facial-recognition-based diagnosis is becoming one of the most promising and novel fields in interdisciplinary medical practice. It accelerates the screening and detection process of diseases, resulting in an earlier start of comprehensive treatment. Though facial manifestations potentiate identification simply from patients’ appearance, diseases with such particular facial characteristics are mostly complex and rare. In traditional diagnosis methods, to be aware of these diseases is the first challenge, especially for doctors who do not have sufficient experience. The second challenge is to select proper inspections and achieve definite analysis of results. Therefore, the effectiveness of AI facial recognition technology has hypothetically given rise to the opportunity for a more time-saving and cost-saving diagnosis path with less interference from empirical error.

However, to date, numerous advancements in algorithms and applications were widely scattered. Few studies summarize or integrate developments and cutting-edge updates in depth or in breadth. Thus, this is the first review on facial-recognition-based applications in disease diagnosis. The aim is to discuss the evolution and classification of technology, and to focus on clinical implications and advantages of facial recognition in diagnosis. This will be helpful to both clinicians and computer scientists in the following ways: to have an overview of current research, to promote medical engineering cooperation, and to stimulate interest in more investigation.

## 2. The Facial Recognition System: Approaches and Algorithms

### 2.1. Image Capture

Static cameras, video cameras, and mobile devices installed with photography software [4] have been used for image capture. After the development of three-dimensional (3D) photography, 3D scanners have also been used to photograph and synthesize facial features [11,12]. The environment setting is mostly in a hospital or a medical organization. In some advanced designs, the application is both patient-side and physiotherapist-side, connected via the cloud network. Images could be taken by patients themselves at home and viewed by doctors through the cloud [13].

Some controllable factors in photography were found to affect the accuracy of facial recognition, including occlusion, low resolution, noise, illumination, pose variation, and expressions [14]. Defects originated from the environment, camera conditions, user’s face conditions, and user-camera positioning [15]. To acquire images with high quality, the process of image capture is standardized. In current studies, clinicians ask patients to expose the entire face and ears; to tidy up hair; to open the eyes and look straight; to close the mouth; to present a neutral and relaxed expression [16,17]. In neurological disorders, patients are asked to perform speech or motor tasks to evaluate their facial neuromuscular function [18]. The position of the camera and patients is fixed in a preset distance and angle to ensure stable illumination intensity. A light source is also required to illuminate the face uniformly. In some research, photos of both sides of the face are taken for more information [3].

### 2.2. Workflow of Facial Recognition Technology

**Figure 1:**
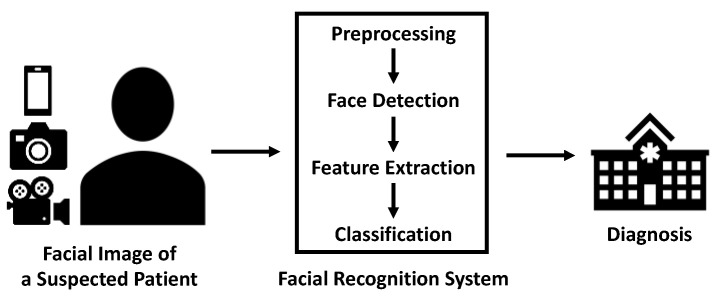
Workflow of facial recognition in disease diagnosis.

After facial images of acceptable quality are obtained, these images are processed in three steps: face detection, feature extraction, and classification (Table 1) [1]. The image is first preprocessed for further normalization. Face detection corresponds to localization of the human face in the image [19]. After detection, facial phenotypes are extracted based on knowledge, statistical analysis, or deep learning [1]. Then, similarity is calculated by matching these features to the database. When the similarity exceeds a certain threshold, the image is classified [20]. In the user’s interface, they input facial images of a suspected patient into the facial recognition system. Then, the automated classifier outputs a categorical variable as to whether the subject is a patient or a healthy control (Figure 1).

In the development stage of the system, both images of patients and controls are collected. Controls are normally age- and sex-matched individuals without the target disease. In some conditions, the sample is divided into a training set and a testing set processed by cross-validation [17,21]. The training set is to establish the algorithm and refine parameters. The testing set is to test the performance and diagnosis accuracy.

### 2.3. Facial Analysis Algorithms

Facial recognition algorithms are categorized into the appearance-based method, the local-feature-based method, and deep learning [14,19]. Classical and frequently used algorithms in disease diagnosis are summarized in Table 1.

#### 2.3.1. Traditional Methods

The appearance-based method, also known as the global feature method, is a model that processes the human face as a whole subject. It extracts global features and matches the holistic face to the database. Not requiring geometry knowledge, the human face is reduced to only a few features or vectors. Principal Component Analysis (PCA), Linear Discriminant Analysis (LDA), Discriminant Common Vectors (DCV), and Independent Component Analysis (ICA) are common algorithms. Support Vector Machine (SVM) is often utilized to accomplish feature discrimination. SVM optimizes the performance of PCA and ICA. The appearance-based method is prone to environmental interference and the patient’s face conditions [1,14,19].

In contrast to appearance-based models, the feature-based method, also known as the local feature method, divides the human face into regions based on biological difference, such as nose, eyes, and mouth. This method has been proven to have higher accuracy. Geometric Features, Local Binary Patterns (LBP), Elastic Bunch Graph Matching (EBGM), Histogram of Oriented Gradients (HoG), Elastic Bunch Graph (EBG), and Hidden Markov Model (HMM) are utilized for computation. However, these algorithms need previous knowledge to select proper facial features in the first step of division, which still remains challenging [1,14,19].

#### 2.3.2. Deep Learning Methods

Neural networks, machine learning, and deep learning have addressed the problem of processing multidimensional data. Probabilistic-Decision-Based Neural Networks (PDBNN) and Radial Basis Function (RBF) are neuronal-network-based algorithms with impressive performance in small datasets. Convolutional Neural Network (CNN) has been prevalently used in facial recognition lately with its improvement in accuracy. Deep learning also makes it possible to eliminate emotional variance and the impact of illumination [1,14,19]. Similar to static images, deep learning algorithms to analyze videos recording facial pathological movements for certain diseases have been developed. Three-dimensional CNN, derived from CNN, captures information in multiple adjacent frames [22]. It is applied to detect neurological diseases with facial dysfunction. Other advanced deep learning models, such as long short-term memory (LSTM) were combined with traditional methods for classification [23].

#### 2.3.3. Mature Software

The mature facial recognition software has been gradually incorporated into clinical development of diagnostic approaches. OpenFace 2.0 is an open-source facial behavior analysis tool available to users and researchers. Its framework includes facial landmark detection, head pose tracking, eye gaze, and facial action unit recognition [24]. Some studies directly built their model based on this software [5,25]. The software enables clinical researchers to focus more on improvement of methods according to disease characteristics.

## 3. The Facial Recognition System: Applications and Advantages

### 3.1. Performance in Varieties of Disease

#### 3.1.1. Endocrine and Metabolic Diseases

Endocrine diseases are characterized by abnormal hormone levels. Patients present metabolic disorders and typical changes in facial features of bones, muscles, and soft tissues. General symptoms of these diseases at the early stage are easily confused with other metabolic syndromes. The gold standard diagnosis process is complex, with multiple instances of hormone testing and imaging examinations [26,27]. High performance of facial recognition in diagnosis has caused it to be considered a fast, accessible screening approach.

Acromegaly Due to increased release of growth hormone, individuals with acromegaly often have a rectangular face, enlargement of the nose and lips, prognathism, and bulging forehead [26]. Various algorithms have been developed to detect the face of acromegaly patients. In 2006, Learned-Miller et al. [28] proposed a 3D morphable model to classify the frontal face into different categories in a semi-automated manner. Forty-nine acromegaly patients and controls were identified at an accuracy rate of 85.7%. Another study in 2013 [29] generated a method based on Gabor wavelet transformations to reduce unwanted noise. They tested it with 57 patients and 59 controls who were gender- and age-matched, reaching an overall correct classification rate of 81%. Gencturk et al. [30] raised a coupled system of local binary patterns (LBP) and Manhattan classifiers, resulting in an accuracy rate of 97%. The introduction of larger datasets and machine learning methods makes the facial recognition system more effective. Kong et al. [31] constructed a dataset of 527 cases of acromegaly and 596 controls. The machine-learning-based system proved effective, with sensitivity and specificity both 96%. Wei et al. [32] enrolled 1131 individuals with acromegaly, and 12,598 normal individuals. The achieved area under the receiver operating characteristic curve (AUC) was 0.9556 and accuracy was 94.79%.

Cushing’s Syndrome Under prolonged exposure to cortisol, Cushing’s syndrome is facially characterized by “moon face” with plethora and acne [33]. Kosilek et al. [34] included 60 female Caucasian Cushing’s syndrome patients in their first study. The automatic face classification system achieved an overall accuracy rate of 91.7%. To eliminate the impact of obesity sequelae, they further designed a body mass index (BMI)-matched sample study [3]. They enrolled controls suspected but ultimately negative for Cushing’s syndrome, and matched the two groups by age, gender, and BMI. The sample size was also increased to 180 patients. The classification accuracy decreased to 61.1% in females and 66.7% in males [33]. Semi-automatic facial nodes analysis was used in their research. In 2020, Wei et al. [32] constructed a facial image database of 814 endocrinally verified instances of Cushing’s syndrome. The deep learning model generated AUC of 0.9647 and accuracy of 95.93%.

#### 3.1.2. Genetic and Chromosome Abnormalities

Genetic and chromosome abnormalities present as systemic syndromes and complications. Hereditary changes directly dysregulate fetal face development or indirectly affect facial phenotype under the abnormal systemic environment. Standard diagnosis methods are genome sequencing and chromosome karyotyping to confirm the abnormalities of genetic materials. This process requires the clinicians’ early awareness of the possibility, techniques for detection and analysis, and patients’ payment of the cost for genetic tests. Regardless of these conditions, facial recognition makes screening possible in clinical practice. Early diagnosis will be beneficial for patients in terms of start of early therapy and lifelong support.

Down Syndrome (DS) DS is the most common chromosome aberration caused by trisomy 21, occurring in 1/800 newborns worldwide [35]. Facial features of DS patients include a flattened face, upward slanting palpebral fissures, epicanthus, small ears, and protruding tongue [36]. In early studies [37,38,39], non-deep-learning methods were proposed for small numbers of samples. In 2014, Zhao et al. [36] designed a hierarchical constrained local model using ICA. This model located the anatomical facial landmarks accurately, achieving accuracy of 96.7% in classifying 50 DS pediatric patients and 80 controls. Deep convolutional neural networks (DCNN) in a larger-scale database of 10,562 subjects were formed by Qin et al. [40]. This model reached 95.87% in accuracy and 97.40% in specificity, demonstrating the potential of AI-based facial recognition for fast detection of genetic syndromes. These systems were trained and tested in Caucasians. In another three studies, Thai neonates [41], Thai children [42], and Congolese children [43] were enrolled as Asian and African subjects. Since DS is globally prevalent and race brings about natural facial variations, there is considerable AI performance in these studies, further indicating its practicability.

Turner Syndrome (TS) TS is a complete or partial loss of one chromosome X that occurs in women. The prevalence is approximately 1/2000 in women. Patients display phenotypic changes in multiple systems. Typical TS face is characterized by epicanthus, deformity of the external ear, micrognathia, high-arched palate, and multiple pigmented nevus [44]. Song et al. [16] first constructed a 68 feature-points model based on endocrinology observations. This computer-aided automatic classification system had an accuracy rate of 84.6%. A facial diagnostic system based on DCNN was developed by Pan et al. [45] later. They also had a larger dataset of 207 TS patients and 1074 female controls. As most of these patients had been photographed several times in their follow-up, the research study designed different photo selecting scenarios to eliminate bias. This system achieved high accuracy with AUC over 0.95. They further conducted a small-sample-size prospective study of two TS patients and 35 controls. The system reached 96.7% in sensitivity and 97.0% in specificity. Photographs in both studies were collected from Chinese subjects.

Genetic Disorders Both DS and TS are chromosomal diseases. Genetic disorders not involving chromosomal abnormalities also have facial characteristics. Efforts have been made to establish facial image analysis in recent years. Facial dysmorphology novel analysis (FDNA) is an automatic face classification framework based on Bayesian networks and LBP [46]. DeepGestalt [4] is a facial image analysis framework using computer vision and deep-learning algorithms incorporated in a smartphone app Face2Gene (FDNA Inc., Boston MA, USA). DCNN was also proposed for congenital adrenal hyperplasia [21]. Method, sample size, and efficacy for eight genetic diseases tested by these novel approaches are summarized in Table 2. All except one study generated accuracy or AUC over 90%, indicating high efficacy of the facial recognition system in diagnosing genetic diseases.

#### 3.1.3. Neuromuscular Diseases

Facial phenotypes are essential manifestations in neuromuscular diseases. AI has been extensively applied in the diagnosis of neurological diseases. Though facial recognition is still limited compared to other applications [54], it has demonstrated impressive prospective benefits in a few diseases.

Facial Paralysis Facial paralysis is loose facial muscle or movement dysfunction due to neuropathy. Its diagnosis depends on the doctor’s subjective ranking scale of facial features and muscle movement [55]. Traditional methods are based on asymmetry extraction of two sides of the face. Video clips of 75 patients and 10 controls were evaluated by a method combining Gabor filter, LBP, and Adaboost classification, yielding an accuracy rate of 60.7% [56]. To increase the objectivity of assessment, deep learning methods have been proposed. Guo et al. [57] raised an end-to-end solution that directly analyzes facial image via fine-tuned DCCN. They collected four facial expression images of 105 patients and 75 controls in the experiment and produced a classification accuracy rate of 91.25%. 3DPalsyNet [58] is a facial palsy grading and motion recognition framework using fully 3D CNN, showing accuracy of 82% in facial palsy and 86% in mouth motions, respectively.

Neurodegenerative Diseases Damage or death of neurons in the central nervous system causes neurodegenerative diseases. Parkinson’s disease (PD) results from insufficient secretion of dopamine. Patients often appear with a masked face. Alzheimer’s disease (AD) is the most common form of dementia. Patients progressively lose memory and thinking skills due to brain atrophy and brain cell death. Amyotrophic lateral sclerosis (ALS) is a rare but severe loss of the motor neurons that control voluntary muscles. Automated static image or movement video analysis has been invented to recognize these diseases [59]. Table 3 shows a summary of data type, sample size, method, and efficacy of studies of PD, AD, and ALS. As a novel non-invasive diagnosis approach, facial recognition shows considerable efficacy.

Real-World and Public Datasets Efforts have also been made to construct datasets from real-world scenes and make them available for sharing. Recently, Zhuang et al. [65] built a “in-the-wild” static image dataset of facial weakness from YouTube, Google Image, and other public repositories. They combined landmarks and intensity features to detect pathological facial asymmetry, which yielded considerable accuracy. Bandini et al. [18] have established an accessible gesture video dataset of oro-facial motion impairment, including post-stroke, amyotrophic lateral sclerosis (ALS), and healthy controls. They incorporated clinical data, manual annotation, and DCCN models. To propel the development of accurate approaches and improvement of automatic identification of neurological disorders from videos and images, more real-world-based and public data are needed.

#### 3.1.4. Other Types of Disease

Acute and Severe Illness In recent years, facial recognition has been applied in acute illnesses for faster screening and patient triage in the emergency room. Forte et al. [66] established a CNN model to distinguish between healthy and simulated acutely ill individuals, yielding sensitivity of 100% and specificity of 42.11%. For severe illnesses, Lin et al. [67] proposed a deep learning model to identify coronary artery disease. In a multicenter cross-sectional study of 5796 patients, this method achieved sensitivity of 0.80, specificity of 0.54, and AUC of 0.730. Zhuang et al. [65] has also built a model to identify the asymmetric face of stroke. These studies represented the potential of an automated facial video- or image-based assessing system to detect acute and severe diseases.

Syndromes without Genetic Abnormality Fetal alcohol syndrome (FAS) results from excess alcohol ingestion during maternal pregnancy. Stereo-photogrammetry was used to measure facial features of 44 FAS subjects in an automated manner [7]. FDNA was further applied in fetal alcohol spectrum disorders, and showed considerable performance compared to manual examination [68]. Chronic fatigue syndrome (CFS) is a complicated disorder characterized by extreme fatigue with an unclarified underlying mechanism. Chen et al. [69] proposed a method based on Gabor wavelet filtering and AdaBoost classification for CFS facial recognition diagnosis. They enrolled 294 CFS volunteers and 297 healthy volunteers in their study and the system reached an average accuracy rate of 89.04% on the training set and 88.32% on the testing set.

### 3.2. Clinical Applications

Automated facial analysis has been incorporated in software, e.g., Face++ [62]. These tools are used for identity recognition, security surveillance, etc. in our daily life. Clinical researchers have been working to develop a similar technology so that the patient’s facial picture could be analyzed in a mobile phone so they could receive a diagnosis in seconds. Face2Gene (FDNA Inc., Boston, MA, USA) is one of the most widely used smartphone apps for facial recognition diagnosis [4]. Trained by 216 different genetic syndromes using 17,106 images of 10,953 subjects, this app has been employed in several studies and has proven efficiency. Auto-eFACE is a facial assessment tool based on deep learning software Emotrics (http://www.sircharlesbell.com/, accessed on 18 May 2022) for unilateral facial paralysis grading and evaluation [70].

Relevant programs have also attracted attention from the National Human Genome Research Institute (NHGRI), part of the National Institutes of Health (NIH). Research with NHGRI has been used to develop software to identify 22q11.2 deletion syndrome, also known as DiGeorge syndrome [71]. This system was trained by images of patients from diverse populations, including Caucasians, Africans, Asians, and Latin Americans. Sensitivity and specificity were both greater than 96.6% for all ethnic groups.

### 3.3. Advantages over Traditional Methods

Facial-recognition-based diagnosis has presented the potential to resolve problems in traditional approaches. Diseases amenable to facial diagnosis are mostly complicated with various but not typical clinical manifestations. Due to difficulties in making a definite diagnosis from general inspection, these diseases are diagnosed with a latent period. For instance, acromegaly and Cushing’s syndrome have a delay of 6 years and 2~6 years, respectively [3]. Since these diseases are relatively rare, clinicians, especially basic-level doctors, require experience and knowledge to recognize them. Differential diagnosis between diseases with similar symptoms is another challenge. Moreover, traditional approaches are complex and both time- and money-consuming. To be compared in these aspects, facial detection is more accurate, informative, and time- and cost-saving.

#### 3.3.1. Accurate and Objective

Accurate Studies have found that when compared to clinicians, the automatic system achieved higher accuracy in identifying the same facial image. In early diagnosis of acromegaly, the computer program achieved 86% over 26% accuracy in physicians [72]. Another acromegaly-detecting system showed higher performance than medical experts and general internists, particularly in patients with moderate features [73]. Further studies invited medical workers of different levels. Chen et al. [74] asked physicians and medical students to complete a web-based test including the same photographs of Turner syndrome used in computer testing. The automatic facial classification system showed higher sensitivity and specificity (*p* < 0.001) than participants. Wei et al. [32] compared their AI-based face classifier of acromegaly and Cushing’s syndrome with medical students, residents, and attending doctors, respectively. The system was more accurate than human doctors.

Objective In addition to clinicians, researchers also compared facial analysis to several diagnostic approaches to explore its practicalities. Pioneer studies in the 2000s compared the automated facial recognition system with manual measurement, demonstrating the objectivity of AI-based diagnosis. In conventional diagnosis of FAS, facial anthropometric measurements made with a hand-held ruler by trained dysmorphologists was thought to be the best approach. A stereo-photogrammetric method was developed to measure the facial dysmorphology of FAS children and showed consistency with experts [7]. Another study developed an automated method to quantify facial motion. Its analysis was consistent with that of manual tracking in facial nerve disorders [8]. For diseases relying on subjective assessment with scales, AI classifiers also proved their accuracy. Studies have been conducted in facial paralysis [57] and AD [64]. Facial-recognition-based diagnostics resulted in similar predictions to the House–Brackmann facial nerve grading system and mini-mental state examination (MMSE) in these diseases, respectively.

#### 3.3.2. Comprehensive and Informative

Comprehensive This novel screening technology not only differentiates patients from healthy individuals, but also provides comprehensive diagnostic possibilities. For each input image, the mobile app Face2Gene (FDNA Inc., Boston, MA, USA) outputs a ranked list of 30 possible genetic disorders, with an accuracy rate of 91% in top 10 diseases [4]. Mishima et al. [75] validated Face2Gene (FDNA Inc., Boston, MA, USA) with Japanese populations and proved its efficacy. Porras et al. [76] have also invented a deep phenotyping screening technology to support early risk stratification at the point of care in global populations.

Informative In addition to classification results of diagnosis, the automatic facial analysis generated much more clinical information. Due to the emergence of 3D technology, facial phenotypes could be quantified more accurately and could act as predictors. In acromegaly, facial features have been elucidated relative to disease severity, progression, and recurrence after surgery. Meng et al. [12] found a few vital variables for disease prediction and gender variation. Guo et al. [77] revealed that insulin-like growth factor 1 (IGF-1) levels were linearly correlated to certain features. In another research study of 668 patients, Fan et al. [78] showed that facial features provided better estimation of transsphenoidal surgical (TSS) responses compared with traditional invasive grading based on pituitary image examination (Knosp Grade).

#### 3.3.3. Improvement of Healthcare System

The convergence of medicine and AI is not only beneficial to patients and clinicians, but also improves the healthcare system [79]. Though direct evidence in the field of facial analysis is insufficient, studies have showed that AI improves workflow and reduces medical errors. The most prominent advantage of AI-based facial recognition diagnosis is the breakdown of the knowledge barrier. Since the size of the dataset far exceeds the upper limit of patients seen by an experienced doctor, it enables doctors at any level to have access to a precise diagnosis. Moreover, as the system can be easily installed in a mobile device and processes images in seconds, it takes much less time than the traditional pathway for diagnosis.

## 4. Future Outlook

### 4.1. Expansion of Database Volume

The sample size affects the performance of machine learning models. It is acknowledged that systems trained by larger datasets have better estimation and less bias. Moreover, facial features vary by age, sex, and race naturally. Demographic influence on the performance of face recognition algorithms has been studied, suggesting female, Black, and younger individuals are more difficult to recognize [80]. For diseases with malformations or dysfunction of the trunk and limbs, simultaneous identification would be essential. Langevin et al. [81] has established PARK for PD diagnosis and subsequent monitoring. This interactive framework asks the patient to complete six motor exercises and one audio task with webcam. Considering that the previous atlas featured only individuals of northern European ancestry, NHGRI launched Atlas of Human Malformations in Diverse Populations (https://research.nhgri.nih.gov/atlas/, accessed on 16 May 2022) in September 2016. This atlas aims to collect photos of physical traits of different inherited diseases around the world. Therefore, in addition to genetic syndromes, comprehensive data of face and physical images covering different groups and various diseases are forthcoming to refine current systems.

### 4.2. Factors Affecting Diagnostic Accuracy

Factors affecting the accuracy of automated facial analysis have been demonstrated such as aging, pose variation, partial occlusion, illumination, and facial expression [14]. Technology is continuously being optimized to reduce the impact of these factors. Clinical scientists also investigated potential confounding factors influencing performance of the facial-recognition-based diagnosis system. Pantel et al. [82] selected genetic disorders with overlapping phenotypic spectra and demonstrated that the growing cohort increased the true positive rate, while ethnicity or sex had no significant effect. Furthermore, only the tip of the iceberg has been discovered in patterns underlying disease severity and phenotypes. To interactively quantify facial classification in disease diagnosis, Wu et al. [83] performed a systematic meta-analysis of 12,557 participants in seven single diseases. They found that the complexity of facial features, defined as Facial Recognition Intensity (FRI), contributed to diagnostic accuracy (*p* = 0.021). Increasing the training size and applying deep learning models will help to improve accuracy of low-FRI diseases. Object’s Complexity Theory (OCT) was hypothetically proposed, as the complexity of the targeted objects determines the complexity of AI processing and plays a vital role in performance of the model. More supportive evidence is needed to reveal the laws behind this.

### 4.3. Integration of Novel Technology

In recent years, 3D photography has begun to contain facial depth information and reduce shape distortion. Corresponding machine learning algorithms have been developed to discriminate between genetic syndromes [11]. Three-dimensional CNN has also been proposed to extract and process motion features [22]. This novel technology broadens the range of identification and interpretation, especially for neuromuscular diseases. Moreover, real-time detection could be added to the system to optimize its clinical use. Facial live detection to capture eye blinking discriminates the human face from a photograph [84]. The elaborate analysis of each organ in the face is also promising. Liu et al. [85] has invented a clinically aided diagnosis system to analyze eye images of ocular myasthenia gravis patients. To combine local feature analysis might yield better performance for the whole face identification.

In addition to facial images, human posture and movement could be identified with deep learning methods for kinematic analysis [86]. Emotion and expression are other essential dimensions in facial recognition technology. Emotional neural networks can detect expressions and improve AI learning and generalization [87]. Automatic pain detection technology was invented for non-communicative patients. It assists caregivers and provides more objective assessments [88]. The automatic emotion annotation system based on 2.5D facial landmarking was also proposed to help people with difficulties in interpreting facial expressions. Social lives of individuals with AD, low vision, and autism spectrum disorder patients’ responses would be improved [89]. Another model to evaluate facial reanimation after facial surgery has also been developed [90].

Meanwhile, a systematic review has been performed to figure out algorithm refinement within a limited sample size [91]. Through analysis of machine learning prediction of autism, this study found that Nested Cross-Validation, train/test split stably produces robust and unbiased estimation regardless of sample size. The state-of-the-art of machine learning will keep bringing about expansion and improvement of application in this field.

### 4.4. Applications beyond Diagnosis

Besides diagnosis and evaluation, facial analysis also has potential for prospective therapy and medical education. Ridha et al. [13] has designed a 3D printed headgear for facial paralysis physical therapy. A Google Machine Learning kit was inputted into a paralysis prediction neural network (PPNN) to predict the percentage of paralysis. Coupled with the headgear, this AI system suggests routine therapy time for physiotherapists. Further tests in real patients and tech convergence are to come. AI-based image analysis has been applied in pathology and radiology education [92,93]. For instance, pathologists generate synthesized images for training. This is also useful for quality control and eliminating perceptual and cognitive bias. In the field of facial recognition, phenotypes could be three-dimensionalized as a model to educate medical students and help understand disease occurrence and development.

### 4.5. From Research to Products

The list of the Food and Drug Administration (FDA)-approved AI image interpretation algorithms is expanding rapidly [79]. Though studies and articles have proliferated over the last decade, only a few research studies have been translated into diagnostic aiding tools. Apps or tools that could be easily installed onto mobile devices, such as Face2Gene (FDNA Inc., Boston, MA, USA), are the most practical. To ensure safety and effectiveness of AI and machine-learning-based software as a medical device, FDA has issued the regulatory framework and an action plan [94]. In the future, the focus will not only be to productize the algorithms, but to eliminate bias and validate performance in real-world clinical scenes.

### 4.6. Privacy and Security

Currently, the human face is a sensitive individual privacy concern. When being asked to take photos of their faces, more patients worry about information leak than before [95]. NHGRI asked the patient to sign a consent form before contributing facial images to their atlas website. Ethical implications of facial recognition technology require more regulations and laws. Security, privacy, autonomy, and democratic accountability are the most considered aspects [96]. In a comparative analysis of regulatory frameworks in the United States, Europe, and the United Kingdom, facial recognition technology will improve when considering data protection impact assessments and human rights impact assessments [97]. Another integrative review by Roundtree et al. synthesized [98] academic studies in the past ten years about ethical issues in facial recognition. In clinical practice, facial images of patients should be taken seriously as the medical record. However, external or internal attacks to the deep learning frameworks could exert a safety threat [99]. Another security concern is the vulnerability to adversarial perturbations, especially in deep neural network (DNN) systems. Defenses against adversarial attacks could be divided into gradient masking, robust optimization, and adversarial example detection [100]. In facial recognition, though evaluations and examinations were made to test existing models, few optimized models have been proposed [101,102]. More efforts should be made to secure the facial classifiers in clinical practice. All in all, more regulations and consensus are forthcoming to raise ethical awareness when using this novel technique in disease diagnosis.

## 5. Conclusions

Facial recognition technology has been developing for decades, but the intersection of facial analysis and disease diagnosis is still an emerging field. In clinical settings, the image capture process is standardized to ensure image quality. Traditional computing methods (appearance-based algorithms, feature-based algorithms) and deep learning have been developed for facial detection. The facial recognition system has showed considerable performance in various types of disease, including endocrine and metabolic disease, genetic and chromosome abnormality, neuromuscular disease, and acute and severe illness. A few software programs have been applied in the clinical practice. Compared to the routine diagnostic approach, facial-recognition-based detection is more accurate, objective, comprehensive, and informative. It also makes it possible to improve healthcare system efficiency. For future perspectives, the facial database volume could be expanded, and factors affecting diagnostic accuracy are to be investigated. Cutting-edge technology could be incorporated into the system to improve its performance. More mature products developed from research are forthcoming. Applications beyond diagnosis are under exploration. Privacy and security are essential ethical problems that need more consideration and regulation. Clinicians and scientists are making continuous efforts to better serve medicine and healthcare.

## Figures and Tables

**Table 1:** Facial Analysis Algorithms.

Category	Algorithm
Appearance-based	Principal Component Analysis (PCA) Eigenface-Based Methods PCA Algorithm Kernel Principal Component Analysis (Kernel PCA) 2D-Image Principal Component Analysis (2D Image IPCA) Linear Discriminant Analysis (LDA) Discriminant Common Vectors (DCV) Independent Component Analysis (ICA) IPCA-ICA Super Vector Machine (SVM)
Feature-based	Geometric Features Local Binary Patterns (LBP) Elastic Bunch Graph Matching (EBGM) Histogram of Oriented Gradients (HoG) Elastic Bunch Graph (EBG) Hidden Markov Model (HMM)
Deep learning	Probabilistic-Decision-Based Neural Networks (PDBNN) Radial Basis Function (RBF) Convolutional Neural Network (CNN)

**Table 2:** Studies of facial-recognition-based diagnosis system for genetic disorders.

Study	Disease	Method	Sample Size	Efficacy
Basel-Vanagaite et al. [46]	Cornelia de Lange syndrome	FDNA	31 cases in training set, 17 cases in testing set	Accuracy = 87% (training), accuracy = 94% (testing)
Latorre-Pellicer et al. [47]	Cornelia de Lange syndrome	Face2Gene	49 cases	Accuracy = 83.7%
Hadj-Rabia et al. [48]	X-linked hypohidrotic ectodermal dysplasia	Face2Gene	136 cases, 717 controls	AUC ≥ 0.98
Liehr et al. [49]	Emanuel syndrome (ES) Pallister-Killian syndrome (PKS)	Face2Gene	59 ES, 70 PKS, 973 controls, 973 others	AUC ≥ 0.98
Amudhavalli et al. [50]	Aymé-Gripp syndrome	Face2Gene	13 cases, 20 controls, 20 DS	AUC = 0.994 (controls), AUC = 0.994 (DS)
Pode-Shakked et al. [51]	Mucolipidosis type IV	Face2Gene	26 cases, 98 controls, 99 others	AUC = 0.822 (controls), AUC = 0.885 (others)
Wang et al. [52]	Kabuki syndrome	Face2Gene	14 cases	Accuracy = 93%
AbdAlmageed et al. [21]	Congenital adrenal hyperplasia	DNN	102 cases, 144 controls	AUC = 92%
Porras et al. [53]	Noonan syndrome (NS) Williams-Beuren syndrome (WBS)	LBP, SVM	286 NS, 161 WBS	Accuracy = 85.68%

Abbreviations and explanations: cases, patients; controls, patients without genetic disorders; others, patients with other genetic disorders; FDNA, facial dysmorphology novel analysis; Face2Gene (FDNA Inc., Boston, MA, USA); AUC, area under the curve; DS, Down syndrome; DNN, Deep Neural Network; LBP, Local Binary Pattern; SVM, Support Vector Machines.

**Table 3:** Facial-recognition-based diagnosis system for neurodegenerative diseases.

Study	Disease	Data	Sample Size	Method	Efficacy
Bandini et al. [60]	PD	Video	17 PD, 17 HC	Intraface tracking algorithm, Euclidean distance, SVM	Difference (*p* < 0.05) between PD and HC
Rajnoha et al. [61]	PD	Image	50 PD, 50 HC	Random Forests, XGBoost	Accuracy = 67.33%
Jin et al. [23]	PD	Video	33 PD, 31 HC	Face++ [62], tremor extraction, LSTM neural network	Precision = 86%
Ali et al. [5]	PD	Video	61 PD, 543 HC	OpenFace 2.0 [24], SVM	Accuracy = 95.6%
Hou et al. [63]	PD	Video	70 PD, 70 HC	HOG, LBP, SVM, k-NN, Random Forests	F1 = 88%
Nam et al. [25]	AD	Video	17 AD, 17 HC	OpenFace 2.0 [24], extract movement coordinates to calculate Spearman’s correlation coefficient	Difference (*p* < 0.05) between AD and HC
Umeda et al. [64]	AD	Image	121 AD, 117 HC	Xception, SENet50, ResNet50, VGG16, and simple CNN with SGD and Adam optimizer	Xception with Adam showed the best accuracy = 94%
Bandini et al. [18]	ALS	Video	11 ALS, 11 HC	AAM, CLM, ERT, SDM, FAN	Accuracy = 88.9%

Abbreviations and explanations: PD, Parkinson’s disease; AD, Alzheimer’s disease; ALS, amyotrophic lateral sclerosis; HC, healthy control; SVM, Support Vector Machines; LSTM, Long Short-Term Memory; HOG, Histogram of Oriented Gradient; LBP, Local Binary Pattern; k-NN, k-Nearest Neighbors; AAM, active appearance models; CLM, constrained local model; ERT, ensemble of regression trees; SDM, supervised descent method; FAN, face alignment network.

## Data Availability

Not applicable.
